# Supplementary material for: Two Phages of the Genera Felixunavirus Subjected to 12 Hour Challenge on Salmonella Infantis Showed Distinct Genotypic and Phenotypic Changes
Source: Viruses. 2019 Jun 27;11(7):586. doi: 10.3390/v11070586 (PMC6669636; doi:10.3390/v11070586)
Supplement: Supplementary file 1 [file viruses-11-00586-s001.pdf]

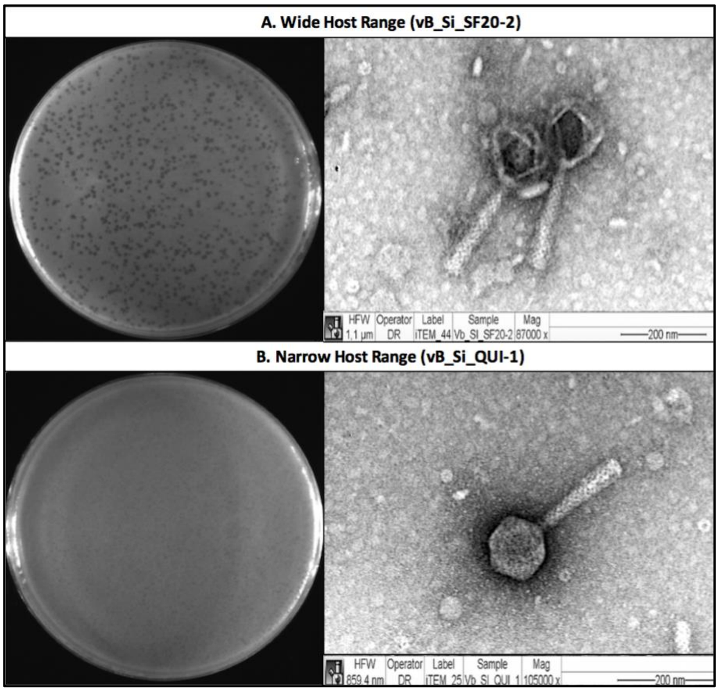

Figure S1. Morphology of lysis plaques and electron microscopy of wide host range and narrow host range phages. Morphology of lysis plaques and electron microscopy of transmission in **(A)** wide host range phage **(B)** narrow host range phage.

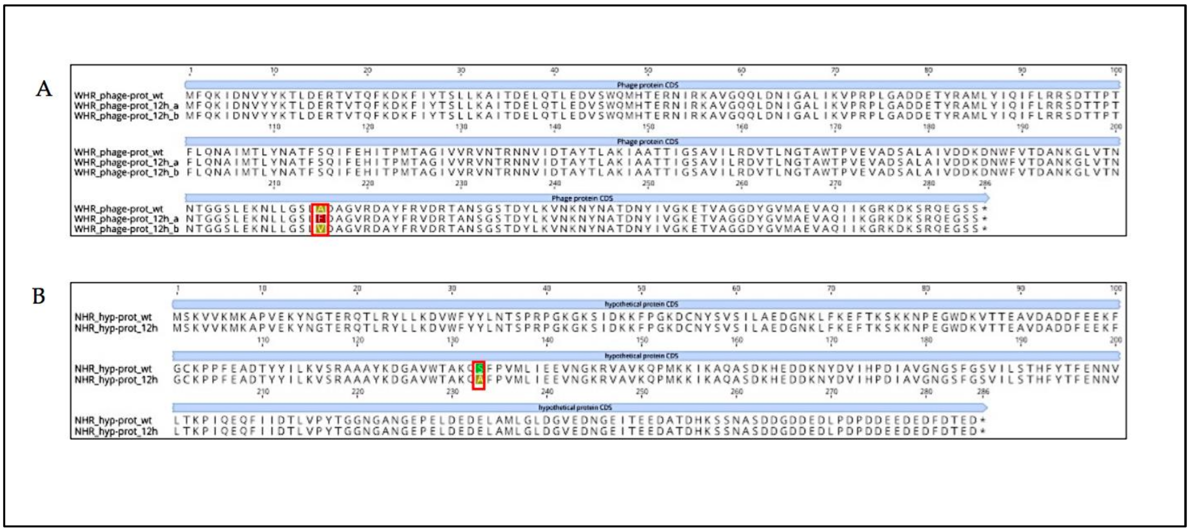

Figure S2. Amino acid alignment of phage protein and hypothetical protein in wild-type phage compared to WHR or NHR phage exposed to *S*. Infantis for 12-h. **(A)** Amino acid alignment of phage protein in wild-type WHR phage and WHR phage exposed to *S*. Infantis for 12-h. Sequences a and b represent two different mutations observed at the same position. **(B)** Amino acid alignment of hypothetical protein in wild-type NHR phage and NHR phage exposed to *S*. Infantis for 12-h. The nucleotide sequence of the gene from the wild type genome was extracted and modified to reflect the mutation, then the wild type and mutation-containing nucleotide sequences were translated and aligned with ClustalW[75] in Geneious Prime[76]. Amino acids labeled with different colors represent differences among sequences of wild-type phages and the 12-h exposed phages. Amino acid changes caused by SNPs are marked in red frames.

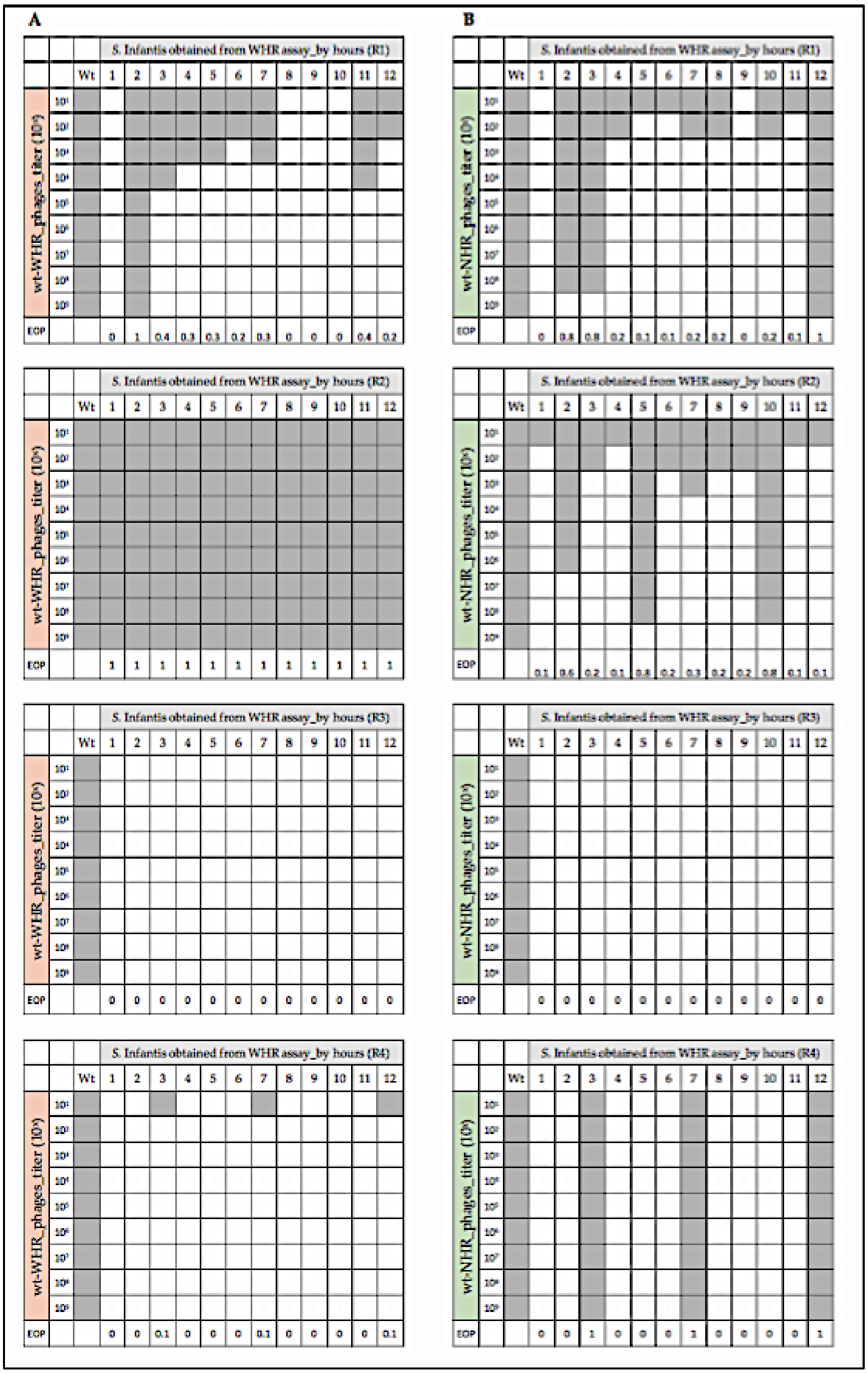

Figure S3. Evaluation of phage sensitivity by efficiency of plating (EOP) on isolated *S.* Infantis *SI* survivors to WHR phage and cross-resistance assays. It shows the titer of the wild type phage on *S.* Infantis obtained from challenge assay. Lysis is marked in grey and no lysis in white. A. Evaluation of phage sensitivity of EOP in *S.* Infantis obtained every hours of wide assay, exposed to wild type (wt)-WHR phage. B. Cross-resistance assay of EOP in *S.* Infantis obtained every hour of wide assay, exposed to wild type (wt)-NHR phage.

A

			S. Infantis obtained from NHR assay_by hours (R1)											
		wt	1	2	3	4	5	6	7	8	9	10	11	12
wt-NHR_phages_titer (10^{x})	10^1													
	10^2													
	10^3													
	10^4													
	10^5													
	10^6													
	10^7													
	10^8													
	10^9													
EOP			0	0	0	0	0	0	0	0	0	0	0	0

			S. Infantis obtained from NHR assay_by hours (R2)											
		wt	1	2	3	4	5	6	7	8	9	10	11	12
wt-NHR_phages_titer (10^{x})	10^1													
	10^2													
	10^3													
	10^4													
	10^5													
	10^6													
	10^7													
	10^8													
	10^9													
EOP			0	0	0	0	0	0	0	0	0	0	0	0

			S. Infantis obtained from NHR assay_by hours (R3)											
		wt	1	2	3	4	5	6	7	8	9	10	11	12
wt-NHR_phages_titer (10^{x})	10^1													
	10^2													
	10^3													
	10^4													
	10^5													
	10^6													
	10^7													
	10^8													
	10^9													
EOP			0	0	0	0	0	0	0	0	0	0	0	0

			S. Infantis obtained from NHR assay_by hours (R4)											
		wt	1	2	3	4	5	6	7	8	9	10	11	12
wt-NHR_phages_titer (10^{x})	10^1													
	10^2													
	10^3													
	10^4													
	10^5													
	10^6													
	10^7													
	10^8													
	10^9													
EOP			0	0	0	0	0	0	0	0	0	0	0	0

B

			S. Infantis obtained from NHR assay_by hours (R1)											
		wt	1	2	3	4	5	6	7	8	9	10	11	12
Wt-WHR_phages_titer (10^{x})	10^1													
	10^2													
	10^3													
	10^4													
	10^5													
	10^6													
	10^7													
	10^8													
	10^9													
EOP			0	0	0	0	0	0	0	0	0	0	0	0

			S. Infantis obtained from NHR assay_by hours (R2)											
		wt	1	2	3	4	5	6	7	8	9	10	11	12
Wt-WHR_phages_titer (10^{x})	10^1													
	10^2													
	10^3													
	10^4													
	10^5													
	10^6													
	10^7													
	10^8													
	10^9													
EOP			0	0	0	0	0	0	0	0	0	0	0	0

			S. Infantis obtained from NHR assay_by hours (R3)											
		wt	1	2	3	4	5	6	7	8	9	10	11	12
Wt-WHR_phages_titer (10^{x})	10^1													
	10^2													
	10^3													
	10^4													
	10^5													
	10^6													
	10^7													
	10^8													
	10^9													
EOP			0	0	0	0	0	0	0	0	0	0	0	0

			S. Infantis obtained from NHR assay_by hours (R4)											
		wt	1	2	3	4	5	6	7	8	9	10	11	12
Wt-WHR_phages_titer (10^{x})	10^1													
	10^2													
	10^3													
	10^4													
	10^5													
	10^6													
	10^7													
	10^8													
	10^9													
EOP			0	0	0	0	0	0	0	0	0	0	0	0

Figure S4. Evaluation of phage sensitivity by efficiency of plating (EOP) on isolated *SI* survivors to NHR phage and cross-resistance assays. It shows the titer of the wild type phage on *S.* Infantis obtained from challenge assay. Lysis is marked in grey and no lysis in white. **A.** EOP in *S.* Infantis obtained every hour of narrow assay, exposed to (wt) wild type-NHR phage. **B.** Cross-resistance assay of EOP in *S.* Infantis obtained every hours of narrow assay, exposed to wild type (wt)-WHR phage.

Table S1. Antigenic somatic formulae (O) and presence of *rfaB* and *rfaK* gene in serovars of *Salmonella enterica* susceptible to wide and narrow host range phages obtained from challenge assays.

**Characteristics of susceptible serovars of both wide and narrow host range phages**			
Serovars (Serogroup)	Somatic antigenic (O) formulae	Presence *rfaB*[1] (ID)[2]	Presence *rfak*[3] (ID)[2]
Typhimurium (Group O:4 [B])	1,4,[5],12	TMV96147.1	ASF66624.1
Virchow (Group O:7 [C_1])	6,7,14	AZH72471.1	AZH72476.1
Oranienburg (Group O:7 [C_1])	6,7,14	THC06534.1	SUH68841.1
Braenderup (Group O:7 [C_1])	6,7,14	Not found[4]	SUH55395.1
Infantis (Group O:7 [C_1])	6,7,14	TMV47934.1	SQI71138.1
Newport (Group O:8 [C_2])	6,8,20	TMM69866.1	AGS27141.1
Corvallis (Group O:8 [C_3])	8,20	TLC03182.1	Not found[4]
Kentucky (Group O:8 [C_3])	8,20	TMW25308.1	KUD18860.1
Javiana (Group O:9 [D_1])	1,9,12	Not found[4]	KUD61483.1
Dublin (Group O:9 [D_1])	1,9,12	TKX13464.1	SUI05380.1
Panama (Group O:9 [D_1])	1,9,12	Not found[4]	SUH76918.1
**Characteristics of susceptible serovars of only wide host range phage**			
Muenster (Group O:3,10 [E_1])	3,{10}{15}{15,34}	**Not found[4]**	KTW46212.1
Agona (Group O:4 [B])	1,4,[5],12	TLA84965.1	RKD15901.1
Saintpaul (Group O:4 [B])	1,4,[5],12	QCW59584.1	KUD72187.1
Heidelberg (Group O:4[B])	1,4,[5],12	TMV96713.1	RFN36928.1
I 4,5,12:i:- (monophasic variant of Typhimurium) (Group O:4 [B])	4,5,12	BBH80090.1	BBH80095.1
Stanley (Group O:4 [B])	1,4,[5],12,[27]	TLC20503.1	SQH42842.1
Choleraesuis (Group O:7 [C_1])	6,7	**Not found[4]**	**Not found[4]**
Mbandaka (Group O:7 [C_1])	6,7,14	RJJ85429.1	RAN13577.1
Montevideo (Group O:7 [C_1] or O:54[5])	6,7,14 or {6,7,14}{54}[5]	TMM93554.1	AJQ76118.1
Enteritidis (Group O:9 [D_1])	1,9,12	TLC90283.1	SUI12981.1

[1](rfaB) gene encoding for the enzyme waaB engaged in the polymerization of core LPS adding UDP-D-galactose lipopolysaccharide

1,6 glucosyltransferase or Lipopolysaccharide 1,6-galactosyltransferase.

[2]GenBank ID: Accession number.

[3](rfak) gene encoding for the enzyme waak engaged in the polymerization of core LPS adding UDP-D-glucose:lipopolysaccharidealpha-1,2 glucosyltransferase or lipopolysaccharide 1,2-N acetylglucosaminetransferase.

[4]Not Found: These genes were not found in the mentioned serovars but have been described for Salmonella enterica.

[5]Factor O:54 is plasmid-controlled. In serovar Montevideo, factors O:6,7,14 are expressed in the absence of O:54. Reference of Somatic antigenic (O) formulae: Grimont et al,[82].
